# Response of Nitrifier and Denitrifier Abundance and Microbial Community Structure to Experimental Warming in an Agricultural Ecosystem

**DOI:** 10.3389/fmicb.2018.00474

**Published:** 2018-03-14

**Authors:** Tatoba R. Waghmode, Shuaimin Chen, Jiazhen Li, Ruibo Sun, Binbin Liu, Chunsheng Hu

**Affiliations:** ^1^Key Laboratory of Agricultural Water Resources, Center for Agricultural Resources Research, Institute of Genetic and Developmental Biology, The Chinese Academy of Sciences, Shijiazhuang, China; ^2^University of Chinese Academy of Sciences, Beijing, China

**Keywords:** warming, irrigation, potential nitrification rate, nitrifier, denitrifier, microbial community

## Abstract

Soil microbial community plays an important role in terrestrial carbon and nitrogen cycling. However, the response of the soil nitrifier and denitrifier communities to climate warming is poorly understood. A long-term field warming experiment has been conducted for 8 years at Luancheng Experimental Farm Station on the North China Plain; we used this field to examine how soil microbial community structure, nitrifier, and denitrifier abundance respond to warming under regular irrigation (RI) and high irrigation (HI) at different soil depths (0–5, 5–10, and 10–20 cm). Nitrifier, denitrifier, and the total bacterial abundance were assessed by quantitative polymerase chain reaction of the functional genes and 16S rRNA gene, respectively. Bacterial community structure was studied through high throughput sequencing of the 16S rRNA gene. Under RI, warming significantly (*P* < 0.05) increased the potential nitrification rate and nitrate concentration and decreased the soil moisture. In most of the samples, warming increased the ammonia-oxidizing bacteria abundance but decreased the ammonia-oxidizing archaea (AOA) and denitrifier (*nirK*, *nirS*, and *nosZ* genes) abundance. Under HI, there was a highly increased AOA and 16S rRNA gene abundance and a slightly higher denitrifier abundance compared with RI. Warming decreased the bacterial diversity and species richness, and the microbial community structure differed greatly between the warmed and control plots. The decrease in bacterial diversity was higher in RI than HI and at the 0–5 cm depths than at the 5–10 and 10–20 cm soil depths. Warming led to an increase in the relative abundance of Actinobacteria, Bacteroidetes, and TM7 but a decrease in Acidobacteria, Alphaproteobacteria, Deltaproteobacteria, Nitrospira, and Planctomycetes. The greater shift in microbial community structure was observed only in RI at the 0–5 cm soil depth. This study provides new insight into our understanding of the nitrifier and denitrifier activity and microbial community response to climate warming in agricultural ecosystems.

## Introduction

The soil microbial community plays an important role in terrestrial nutrient cycling; many biological processes involved in nitrogen (N) cycling in terrestrial ecosystems are altered due to climate warming (Mosier, 1998; Rustad et al., 2001), and these changes are likely to result in altered plant productivity and atmospherically active gases (Mosier, 1998; Barnard et al., 2006). However, due to the complexity of the microbial community in soil, how climate warming affects the activity, abundance, and structure of microbial community is poorly understood (Rui et al., 2015).

Warming can directly affect soil bacterial physiology and indirectly affect microbial activity through changing plant and soil properties (Rui et al., 2015). For example, an increase in temperature may lead to a shift in community structure and can enhance the predominance of thermally adapted microorganisms (Bradford et al., 2008). Previous long-term warming experiments have shown that warming alone (Zhou et al., 2012; Rui et al., 2015) and in combination with precipitation (Castro et al., 2010) could alter the microbial community structure in the soil. Moreover, warming is responsible for moderate natural drought and decreased microbial diversity, with significant changes in community composition (Sheik et al., 2011). Xu et al. (2016) reported that simulated warming and drying conditions are responsible for altering the nitrifier and denitrifier community in vegetable soil. However, these field experiments mainly focused on grasslands, grass prairies, alpine forest, and vegetable soil to explain how the community composition of N cycling microorganisms was altered by simulated warming. In addition, the feedback response of microorganisms involved in nitrous oxide (N_2_O) emission caused by warming and drought differed between various ecosystems (Singh et al., 2010). No study is available regarding the effects of simulated warming on communities involved in N cycling (i.e., nitrifier and denitrifier) and microbial community structure under an agricultural ecosystem, especially intensely fertilized wheat fields in the North China Plain. Understanding the effects of climate warming on the abundance of nitrifiers and denitrifiers, which carry out key processes such as nitrification and denitrification, is important because these processes influence soil inorganic N concentrations, nitrate leaching, and the production of N_2_O (Barnard et al., 2006).

In the alpine forest and polar regions, warming can increase the soil moisture content due to glacier and permafrost melting (Walther et al., 2002) and thereby can enhance the activity of microbial communities involved in nitrification and denitrification. However, these observed changes are different from those in upland agricultural ecosystems, where climate warming is often accompanied by decreased soil moisture (Liu et al., 2016). Soil-warming experiments can offer an opportunity to elucidate the response of a microbial community to climate warming. Previous study from our group has reported that the experimental warming decreased N_2_O emissions (Liu et al., 2016), possibly due to drier soil conditions which may be unfavorable for denitrifying activity. However, that study only provides evidence regarding the impact of climate warming on N_2_O emission from the soil; the response of nitrifiers and denitrifiers involved in N_2_O emission and microbial community composition to climate warming was not investigated.

In the present investigation, we aimed to elucidate the response of nitrifier and denitrifier abundance and microbial community structure to climate warming in an agriculture ecosystem. We hypothesized that (i) nitrifier and denitrifier abundance would respond differently to climate warming, as an increase in soil temperature would increase nitrifier abundance and decrease denitrifier abundance, and (ii) the microbial community structure would respond differently between regular irrigation (RI) and high irrigation (HI), as well as at different soil depths, to climate warming. To test these hypotheses, sampling was performed from the ongoing long-term (for 8-year) warming experiment with wheat cultivation in RI and HI plots at different soil depths. We assessed the nitrifier and denitrifier abundance by quantitative polymerase chain reaction (Q-PCR) and the microbial community structure by 16S rRNA gene sequencing.

## Materials and Methods

### Site Description

The soil sampling site was located at the Luancheng Experimental Farm Station (37° 53′N, 114° 41′E, 50 m above sea level) of the Chinese Academy of Sciences, Hebei Province, China. Detailed information about the experimental site has been described previously (Liu et al., 2016). In brief, this long-term warming experiment began in 2008. Six pairs of infrared heaters (2 m × 0.02 m in size) with a rated power of 1000 W were installed 2 m above the ground at the center of six plots. The plot allocation treatments were randomized. The plot size was 4 m × 4 m, and the effective radiation area was 2 m × 2 m. Another six pairs of the same framework and heaters without power were placed in plots next to the heating plots at a distance of approximately 1 m to mimic the shadow effect of the heater frames. The crop under cultivation was winter wheat. The soil at the experimental site was classified as sandy loam with soil pH 8.1 (1:2.5 with H_2_O), organic matter 15.1 g kg^-1^, and total N 1.1 g kg^-1^ at a 0–20 cm soil depth.

### Design of the Field Study and Soil Sampling

An experiment was conducted with simulated warming (temperature increased approximately 1.5 °C on average over 7 years at a 5 cm soil depth) and its control (no warming) under RI and HI (i.e., the treatments were designated as warmed and control under RI and HI; Liu et al., 2016). The fertilizer dose (N fertilizer, 315 kg N ha^-1^ year^-1^; P fertilizer, 65 kg P ha^-1^ year^-1^) used in this experiment was the same for all treatments, as reported previously (Liu et al., 2016). In case of irrigation, RI and HI plots were irrigated with 60 and 90 mm, respectively, at the same time. Irrigation was applied one time before the soil sampling (first week of April 2016). The high irrigation treatments included in this study are based on the conclusion from our previous field experiment, which states that high irrigation overrides the warming impact on denitrifying activity (Liu et al., 2016).

Soil sampling was performed at 0–5 (5), 5–10 (10), and 10–20 cm (20 cm) soil depths within effective radiation area in triplicates (April 2016). Three soil cores were randomly taken from each triplicate plot by auger (3.2 cm diameter) and mixed together to get a composite sample. The soil samples were transported to the lab in an icebox, sieved through a 2 mm sieve and stored at 4 °C for biochemical analyses and -80 °C for gene abundance and microbial community composition analyses.

### Soil Temperature, Moisture, and Mineral N Concentration

T-type thermocouple lines were placed in the soil at the center of all plots to automatically monitor the soil temperature at 0–5 cm every hour, which was recorded by a data logger (CR 10X, Campbell, CA, United States). The volumetric soil water content at 0–5 and 10–20 cm depths in each plot was measured manually by time-domain reflectometry.

Five grams of soil were extracted with 50 mL of 2 M KCl and shaken at 150 rpm for 30 min. The filtered solution was then analyzed for ammonium (NH_4_^+^) and nitrate (NO_3_^-^) using a spectrophotometer (UV-2450, Shimadzu, Japan). The NH_4_^+^ concentration was estimated by indophenol blue method (Page et al., 1982), 8 mL of filtrate was transferred to the 50 mL glass tube, and then 5 mL of phenol–nitroprusside solution and 5 mL of alkaline hypochlorite solution were added, mixed thoroughly, and waited for 1 h at room temperature to develop a blue color. The optical density was measured at 625 nm. For NO_3_^-^ estimation, filtrate solution was directly used to measure the absorbance at 210 nm (A_220_) and 275 nm (A_275_) wavelength. The concentration of NH_4_^+^ and NO_3_^-^ in the sample was calculated by plotting against a standard curve.

### Potential Nitrification Rate

The potential nitrification rate (PNR) was used in the study as an index for the size of active nitrifier populations in the soil. In brief, for each sample, three subsamples (5 g of fresh soil) were incubated in 50 mL falcon tubes containing 20 mL of phosphate buffer solution including 1 mM (NH_4_)_2_SO_4_. Potassium chlorate (KClO_3_) was added to the tubes at a final concentration of 10 mM to inhibit the nitrite (NO_2_^-^) oxidation. The suspension was incubated in the dark at 25 °C for 24 h, and nitrite was extracted with 5 mL of 2 M KCl. After filtration, the optical density of the supernatant was analyzed for the presence of NO_2_^-^ at 540 nm with N-(1-naphthyl) ethylenediamine dihydrochloride (Kurola et al., 2005). PNR was calculated as the linear accumulation in concentrations of NO_2_^-^ between time 0 and 24 h.

### Soil DNA Extraction

Soil samples from all treatments were selected for functional gene quantification by Q-PCR and microbial community structure analysis through 16S rRNA gene sequencing. Soil total nucleic acids were extracted using an E.Z.N.A.^®^Soil DNA Kit (Omega Bio-tek, Inc., Norcross, GA, United States) according to the manufacturer’s instructions. The quality and quantity of the extracted DNA were examined with agarose gel (1%) electrophoresis and a NanoDrop spectrophotometer (NanoDrop ND-2000c Technologies, Inc., Wilmington, DE, United States). Extracted DNA was stored at -20 °C until further analysis.

### Quantitative PCR Assay of Functional Genes

Quantitative polymerase chain reaction was performed to quantify 16S rRNA gene and functional genes involved in nitrification (*amoA* for bacteria and archaea) and denitrification (*nirS*, *nirK*, and *nosZ*). The *amoA*, *nirK*, *nirS*, *nosZ*, and 16S rRNA genes were quantified using primers as follows: amoA-1F/amoA-2R for bacterial *amoA* (Tourna et al., 2008; Jin et al., 2010), Arch_amoAF/Arch_amoAR for archaeal *amoA* (Francis et al., 2005), F1aCu/R3Cu for *nirK* (Hallin and Lindgren, 1999), cd3aF/R3cd for *nirS* (Michotey et al., 2000; Throback et al., 2004); nosZ-F/nosZ-1622R for *nosZ* (Kloos et al., 2001; Throback et al., 2004), and 1369F/1492R for 16S rRNA gene (Suzuki et al., 2000). Standard curves were constructed using a 10-fold series dilution of the plasmids for seven gradients carrying the respective target genes. The Q-PCR reaction was performed in a 25 μL volume, containing 2 × SYBR Premix Ex Taq (Takara Biotech, Dalian, China), 1 μM of each primer (for functional genes), 2 μM of each primer and 3 μM of probe for 16S rRNA gene, and 1 μL template DNA (20 ng μL^-1^). The Q-PCR program consisted of an initial cycle of 95 °C for 2 min, 40 cycles of 30 s at 95 °C for denaturation (15 s for 16S rRNA gene), 40 s for annealing (53/60 °C for archaeal/bacterial *amoA*, 57 °C for *nirK*, 56.8 °C for *nirS*, 59 °C for *nosZ* and 60 s, 56 °C for 16S rRNA gene), 30 s at 72 °C for extension, and 10 s at 85 °C for collection of the fluorescent signals. Melting curves were generated for functional genes with continuous fluorescence acquisition from 57 to 95 °C at the rate of 0.5 °C per 10 s. After Q-PCR, the gene copy numbers were normalized by the amount of soils based on the dilution rates and the volumes of the DNA used for Q-PCR.

### 16S rRNA Gene Amplicon Sequencing

Microbial community structure was analyzed through sequencing of the 16S rRNA gene of samples from all treatments and all soil depths. Bacterial DNA was amplified with a set of primers targeting hypervariable V3–V4 region (approximately 460 bp) of 16S rRNA gene with attached overhang adapters (FwOvAd-341F: TCGTCGGCAGCGTCAGATGTGTATAAGAGACAGCCTACGGGNGGCWGCAG; ReOvAd-785R: GTCTCGTGGGCTCGGAGATGTGTATAAGAGACAGGACTACHVGGGTATCTAATCC; Yasir et al., 2015). In a 25 μL volume, reaction mixtures contained 2 × premix Ex Taq^TM^ (Takara Biotechnology, Dalian, China), 5 μM of each primer, and 1 μL DNA template (20 ng μL^-1^ concentration). The reaction conditions were an initial cycle of 95 °C for 3 min; 23 cycles of 30 s at 95 °C, 30 s at 55 °C, and 30 s at 72 °C; and a final extension at 72 °C for 10 min. The PCR products were visualized on agarose gels to confirm successful amplification and then purified with AMPure XP beads (Beckman Coulter, Inc., Brea, CA, United States) to remove residual primers and primer dimers following the manufacturer’s protocol. Then, using a subsequent eight-cycle PCR, Illumina sequencing adapters and dual-index barcodes were added to each amplicon. After purification on AMPure beads, the libraries were then normalized according to the Nextera XT (Illumina) protocol. The pooled samples were sent to Shanghai Jiao Tong University, Shanghai, China, and sequenced on a MiSeq platform (Illumina, San Diego, CA, United States).

### Bioinformatics Analysis

The quality of the sequences was inspected with the fastQC program^1^. The paired-end reads were merged using FLASH (version 1.2.11) (Magoč and Salzberg, 2011) with the default settings, except that the maximum overlap length was set to 170. The low-quality merged sequences were then removed using fastx_toolkit software^2^, and only the sequences with more than 80% of the bases that had quality scores higher than 20 were kept. Any sequences with ambiguous bases (N) and sequences outside 414–506 bp (460 ± 10%) in length were discarded for further analysis. Then, the sequences were pooled in one file and input into the Quantitative Insights into Microbial Ecology (QIIME) software suite. The subsampled open-reference workflow was used for Operational Taxonomic Unit (OTU) classification and taxonomy assignment, and OTU picking was performed using uclust (Edgar, 2010) with the default cutoff value (97%). The OTU table was subsampled (rarefied) and the alpha diversity (Shannon–Wiener index) was calculated based on the rarefied OTU tables (Magurran, 1988). The rarefication curves were plotted and presented as Supplementary Figure 2. Principal coordinate analysis (PCoA) was performed using the weighted UniFrac distance matrix between the samples in QIIME pipeline. Sequencing data were deposited into the European Nucleotide Archive under the accession number PRJEB22187.

### Statistical Analyses

Statistical analyses were conducted with Statistix 8.1 and SPSS20.0 software. Analysis of variance (one-way ANOVA), Tukey’s honestly significant difference (HSD, at *P* < 0.05), and unpaired *t*-test (*P* < 0.05) were performed to assess the significant effect of warming on soil physico-chemical parameters, abundance of functional genes, total bacterial gene, and microbial community structure among the treatments and at all soil depth. A two-way ANOVA analysis and HSD (at *P* < 0.05) analysis were performed to assess the main and interactive effect of warming and irrigation on abundance of functional genes and total bacterial gene. SPSS20.0 was used to assess the Pearson’s correlation between PNR, nitrate concentration, and gene abundance [ammonia-oxidizing bacteria (AOB) and ammonia-oxidizing archaea (AOA)].

## Results

### Soil Temperature, Moisture, and Mineral N Concentration

Warming increased the soil temperature by 1.6 °C in RI and 0.8 °C in HI (**Table 1**). In addition, warming decreased the soil moisture content at 5 and 20 cm soil depths compared to the control plots, and the decrease in soil moisture was higher in the RI treatment than in the HI treatment (**Table 1**).

**Table 1:** Effect of warming on the monthly average value of soil temperature and moisture under the regular irrigation (RI) and high irrigation (HI) treatments.

	RI		HI	
Soil depth (cm)	Warmed	Control	Warmed	Control
**Soil temperature (°C)**				
5	12.7 ± 0.74a	11.1 ± 0.44a	12.4 ± 0.55A	11.6 ± 0.65A
**Soil volumetric moisture (%)**				
5	11.1 ± 0.94a	13.6 ± 1.7a	13.1 ± 1.05A	14.7 ± 1.12A
20	10.2 ± 1.29a	12.4 ± 1.75a	12.4 ± 1.27A	14.1 ± 2.35A

*Identical letters in the same row indicate no significant difference at *P* < 0.05 [Tukey’s honestly significant difference (HSD) *post hoc* test]. Values are expressed as the means and standard errors (*n* = 3)*.

Warming increased the NH_4_^+^ concentrations at all soil depths than in control, but this increase was not significant. Warming also increased NO_3_^-^ concentrations in both RI and HI, but the increase was significant (*P* < 0.05) only in RI (**Table 2**). Moreover, the concentration of mineral N was higher at the 5 cm depth and decreased with soil depth.

**Table 2:** Effect of warming on soil NH_4_^+^, NO_3_^-^ concentrations and potential nitrification rate (PNR) activity under RI and HI at different soil depths.

	RI		HI	
Soil depth (cm)	Warmed	Control	Warmed	Control
**NH_4_^+^ (mg-N kg^-1^)**				
5	2.69 ± 0. 10a	2.10 ± 0.02b	4.57 ± 0.26A	4.67 ± 0.12A
10	1.43 ± 0.13a	1.35 ± 0.20a	2.02 ± 0.29A	1.73 ± 0.03A
20	1.42 ± 0.18a	1.10 ± 0.09a	1.34 ± 0.03A	1.28 ± 0.03A
**NO_3_^-^ (mg-N kg^-1^)**				
5	139.8 ± 3.73a	57.4 ± 4.98b	91.2 ± 9.03A	80.4 ± 6.47A
10	126.3 ± 6.95a	43.3 ± 4.19b	74.9 ± 15.6A	61.9 ± 5.07A
20	117.9 ± 2.80a	38.5 ± 3.19b	57.2 ± 7.43A	45.3 ± 1.0A
**PNR activity (mg of NO_2_ kg^-1^ soil day^-1^)**				
5	18.2 ± 0.93a	12.6 ± 2.44b	13.3 ± 0.30A	12.0 ± 0.42A
10	17.7 ± 3.86a	7.88 ± 2.54b	20.2 ± 2.32A	18.6 ± 0.23A
20	19.6 ± 1.17a	11.4 ± 3.62a	18.7 ± 0.91A	17.8 ± 0.10A

*Different letters in the same row indicate a significant difference at *P* < 0.05 (Tukey’s HSD *post hoc* test). Values are expressed as the means and standard errors (*n* = 3)*.

### Potential Nitrification Rate and Abundance of Nitrifiers and Denitrifiers

**FIGURE 1:**
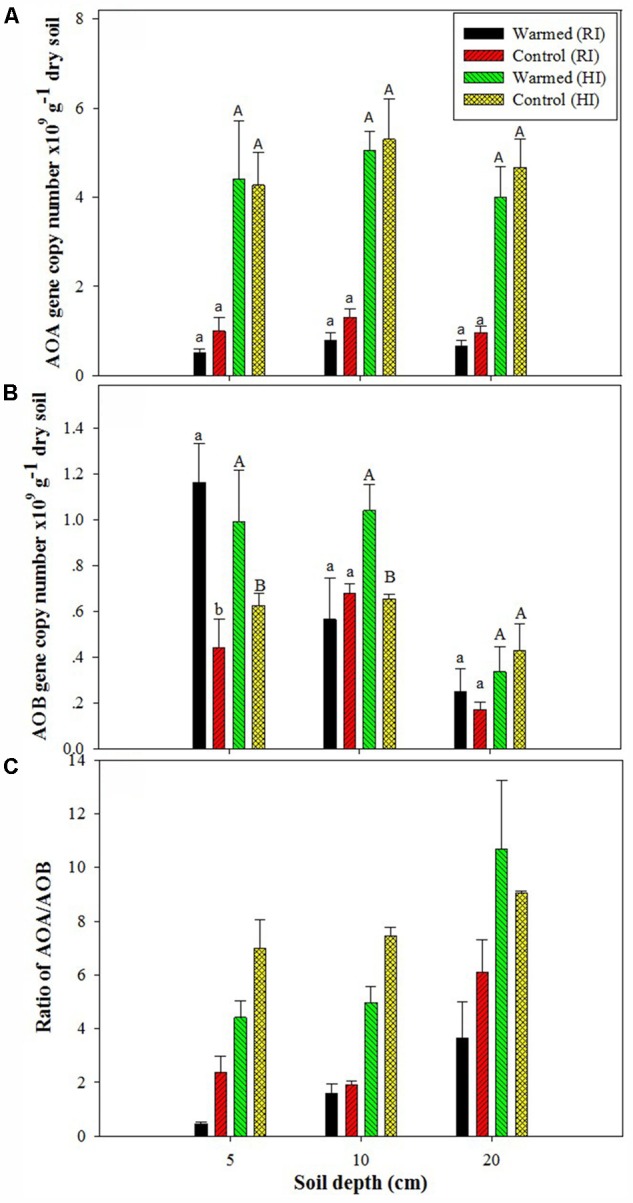
Effect of warming on ammonia-oxidizing archaea (AOA) **(A)**, ammonia-oxidizing bacteria (AOB) gene copy number **(B)**, and AOA/AOB ratio **(C)** under the regular irrigation (RI) and high irrigation (HI) treatments at different soil depths. Different letters indicate a significant difference at *P* < 0.05 (unpaired *t*-test). The error bar indicates the standard error of the mean (*n* = 3).

The PNR was higher in warmed than in control plots at all soil depths, but a significant difference (*P* < 0.05) was observed only in RI (**Table 2**). AOA abundance was slightly lower in warmed plots compared with control plots but was statistically at par (**Figure 1A**) in both irrigation treatments; however, the abundance was more than twofold higher in HI than in RI. In contrast, the AOB abundance was significantly (*P* < 0.05) higher in warmed than in control plots at a 5 cm soil depth in RI and at 5 and 10 cm at HI; there was no significant difference between control and warmed plots at the 20 cm soil depth (**Figure 1B**). AOB abundance was higher in 5 and 10 cm soil than at 20 cm, whereas AOA did not show any decrease in abundance with soil depth. The ratio of AOA to AOB decreased in warmed plots compared with control plots at all soil depths, except at 20 cm in HI (**Figure 1C**). PNR (*r^2^* = 0.74, *P* < 0.01) and nitrate (*r*^2^ = 0.82, *P* < 0.001) showed a positive correlation with AOB abundance, whereas PNR (*r*^2^ = -0.59, *P* < 0.05) exhibited a negative correlation with AOA. Two-way ANOVA analysis showed that warming had a significant correlation with AOB abundance alone (*P* < 0.001) and with irrigation (*P* < 0.05) at the 5 cm soil depth; however, the AOA abundance had a significant (*P* < 0.001) correlation only with irrigation at all soil depths, and there was no significant correlation between warming and AOA abundance (Supplementary Table S1).

**FIGURE 2:**
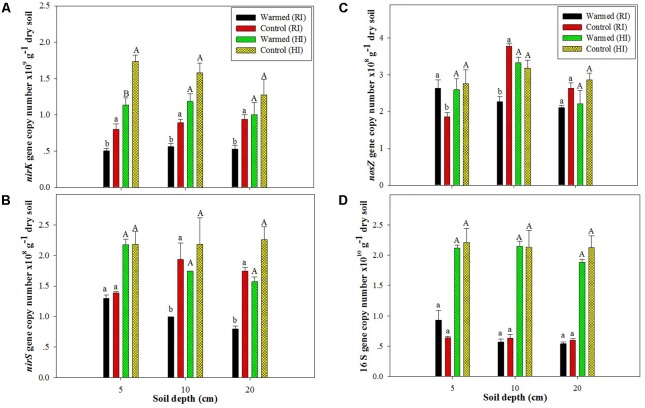
Effect of warming on the abundance of *nirK* **(A)**, *nirS* **(B)**, and *nosZ* **(C)** and on the 16S rRNA gene **(D)** at different soil depths under RI and HI. Different letters indicate a significant difference at *P* < 0.05 (unpaired *t*-test). The error bar indicates the standard error of the mean (*n* = 3).

In the case of denitrifying genes, we observed a significant impact of soil warming on the *nirK*, *nirS*, and *nosZ* gene abundance. The abundance of the *nirK* (**Figure 2A**) and *nirS* (**Figure 2B**) genes was higher in control than in warmed plots at all soil depths and in both irrigation treatments, but the significant (*P* < 0.05) decrease was mostly observed under RI. The *nosZ* abundance was decreased in warmed compared with control plots at 10 and 20 cm soil depths under RI (**Figure 2C**). The relative abundance (normalized to total 16S rRNA gene copies) of functional genes showed a similar trend with denitrifying gene abundance in warmed and control plots (Supplementary Figure 1). Warming and irrigation alone had a significant effect on *nirS*, *nirK*, and *nosZ* abundance, and there was no interactive effect of warming with irrigation on these genes, except for *nirS* and *nosZ* at 20 and 10 cm, respectively (Supplementary Table S1). Warming had no significant effect on 16S rRNA gene abundance under both irrigation treatments, but the higher irrigation increased 16S rRNA gene abundance by more than twofold compared with RI (**Figure 2D**). Irrigation had a significant correlation with 16S rRNA gene abundance at all soil depth (*P* < 0.001); however warming alone and with irrigation had no significant correlation with 16S rRNA gene abundance (Supplementary Table S1).

### Assessment of the Microbial Diversity and Community Structure

**FIGURE 3:**
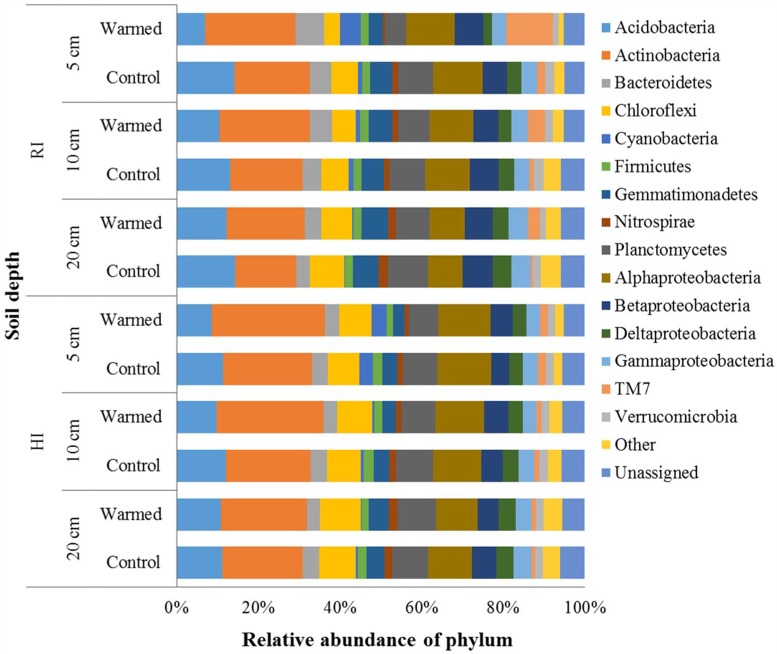
Relative abundance of the dominant bacterial phyla in warmed and control plots at different soil depths under RI and HI irrigation.

In total, 2,751,268 sequences were generated, resulting in 21,343–57,433 sequences per sample. Quality control steps removed around 55% low-quality reads and ended up with 1,224,231 high-quality reads, which were analyzed using QIIME pipeline. After OTU picking, the singletons were removed, and then the OTU table was rarefied to have 12,071 sequences in each sample. In the control and warmed plots, the Proteobacteria, Actinobacteria, and Acidobacteria were the most abundant phyla followed by Planctomycetes, Chloroflexi, Bacteroidetes, and Gemmatimonadetes, and minor sequences were related to the Firmicutes, TM7, Verrucomicrobia, and Nitrospirae (**Figure 3**). Soil warming strongly influenced the abundance of bacterial taxa and a large proportion of phyla were significantly responded to warming in RI treatment (especially at 5 cm soil depth); however, some phyla, such as Acidobacteria, Actinobacteria, Firmicutes, and Gemmatimonadetes, showed significant differences in their relative abundance between warmed and control under HI treatment. We observed a significant increase in the relative abundance of Actinobacteria (*P* < 0.05), which was compensated by a tendentious decrease in Acidobacteria and Proteobacteria (**Figure 3** and Supplementary Table S2). Warming significantly (*P* < 0.05) increased abundance of Actinobacteria under both irrigation treatments except at the 20 cm soil depth under HI; while TM7 increased and Verrucomicrobia decreased significantly (*P* < 0.05) in relative abundance in warmed plot compared to control plot under RI at all soil depths. Order Actinomycetales (Actinobacteria) also showed higher (*P* < 0.05) relative abundance in the warmed plots than in control plots at all soil depths under both irrigation treatments, except at the 20 cm soil depth under HI (Supplementary Table S2). However, Acidobacteria showed a lower relative abundance in warmed plots compared with control plots under both irrigation treatments, but significant decrease was observed in 5 cm soil depth. Gemmatimonadetes, Nitrospirae, and Planctomycetes showed significantly lower relative abundance in warmed compared with control plot at 5 cm soil depth under RI treatment. Firmicutes and Gemmatimonadetes showed a significantly lower relative abundance in warmed compared with control plots under HI at most of the soil depths (Supplementary Table S2). Class Solibacterales (Acidobacteria) also showed a lower abundance (*P* < 0.05) in warmed compared with control plots under both irrigation treatments except at 20 cm where the differences were not significant. Nitrospirales (Nitrospirae) showed a significantly (*P* < 0.05) lower abundance in warmed compared with control plots at the 5 cm in RI and the 5 and 20 cm soil depths in HI treatment. Among the Proteobacteria phyla, relative abundance of Alpha-, Gamma-, and Delta-Proteobacteria was generally lower in warmed plot than control plots. Order Rhodospirillales (Alphaproteobacteria) and Syntrophobacterales (Deltaproteobacteria) showed a lower relative abundance in warmed compared with control plots under RI (significant at 5 cm soil depth). Most of the significant differences in the relative abundances occurred under RI. This meant that the community structure changes caused by warming could be override by high rate irrigation. Soil depth was also influencing the taxon relative abundance, larger proportion of taxa at the 5 cm soil depth (i.e., surface layer) was significantly influenced by warming compared with the 10 and 20 cm soil depths under RI, while the trend was not as obvious in HI (**Figure 3** and Supplementary Table S2).

**FIGURE 4:**
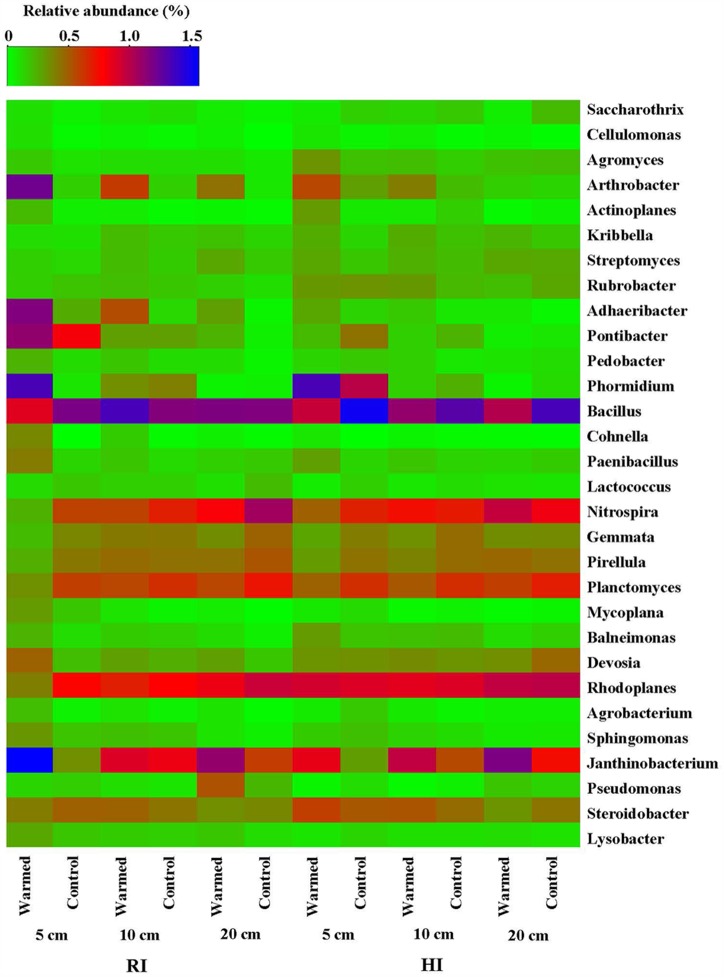
Heatmap of the bacterial distribution of different communities from warmed and control samples at the genus level (most abundant genera either increased or decreased in response to warming were selected). The row represents the relative abundance of each bacterial genus, and the column stands for each sample at different soil depths under RI and HI treatment. The relative abundance of each bacterial genus is depicted by color intensity with the legend indicated at the top of the figure. The relative abundance for each genus in different samples is colored in shades of green (low relative abundance) to red and purple to blue (high relative abundance).

The heatmap of the microbial community displays the relative abundances of dominant bacterial genera either increased or decreased in response to warming under RI and HI at different soil depths (**Figure 4**). A pairwise comparison showed that the relative abundance of genera was strongly influenced by warming and a large proportion of genera were either increased or decreased significantly in relative abundance in RI (especially at 5 cm soil depth) when compared with HI treatment (**Figure 4** and Supplementary Table S2). *Arthrobacter*, *Rubrobacter*, *Cellulomonas*, *Cohnella*, *Mycoplana*, *Janthinobacterium*, and *Lysobacter* were significantly enhanced by warming compared with the control under RI treatment, while *Arthrobacter*, *Rubrobacter*, *Cohnella*, and *Janthinobacterium* were significantly enhanced by warming under HI. However, some genera such as *Nitrospira*, *Rhodoplanes*, *Planctomyces*, and *Gemmata* were decreased in relative abundance by warming compared with the control under RI, and *Bacillus*, *Gemmata*, and *Pseudomonas* (except at 20 cm soil depth) showed a similar response to warming under HI. These results suggested that the significant change in abundance of most bacterial communities in response to soil warming was observed under the RI and was mainly limited to the 5 cm soil depth.

**FIGURE 5:**
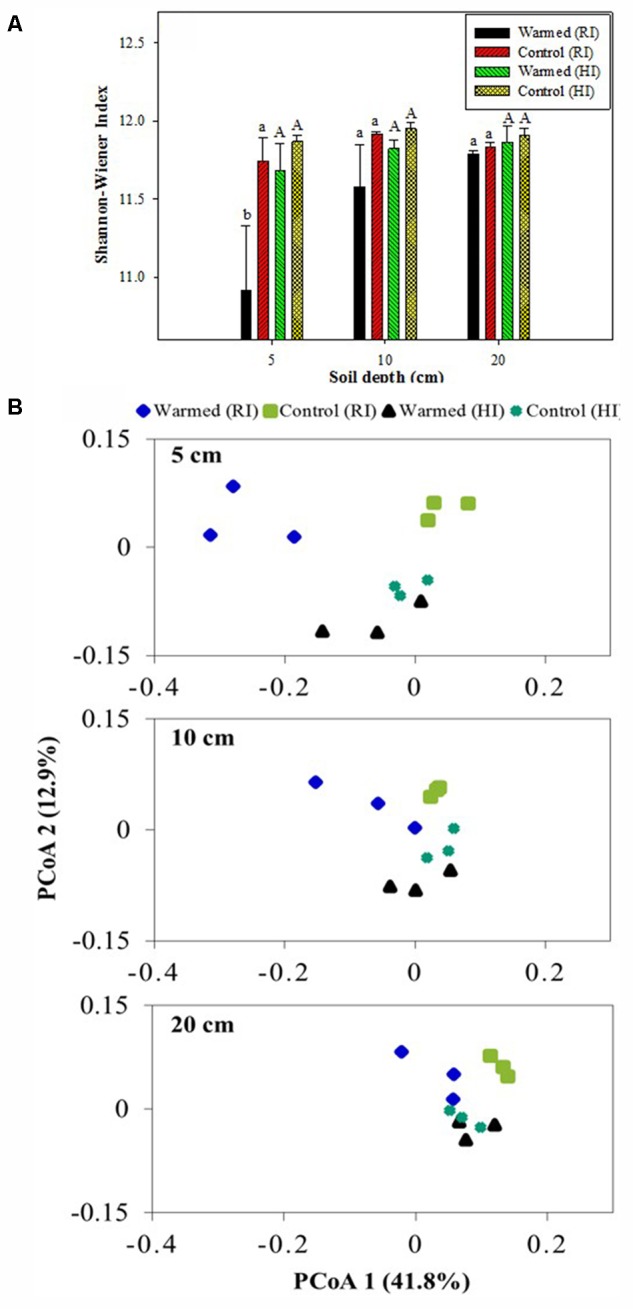
Shannon-Wiener index **(A)** and weighted UniFrac principal coordinate analysis (PCoA, **B**) of the bacterial communities based on OTUs of the 16S rRNA gene from the warmed and control plots under RI and HI at different soil depths. One PCoA ordination was performed for all treatments, but the three depths (5, 10, and 20 cm) are shown separately for clarification. Identical letters indicate no statistically significant difference at *P* < 0.05 (Tukey’s HSD *post hoc* test). The error bars indicate the standard errors of the means (*n* = 3).

Shannon–Wiener and Chao1 indexes were calculated to assess the bacterial diversity and richness. Warming decreased the bacterial diversity compared with the control, and the decrease was much higher in RI than HI; furthermore, the decrease in diversity was significant (*P* < 0.05) at the 5 cm soil depth in RI (**Figure 5A**). The warmed plot had a lower richness compared to the control, with higher OTU number in control than in warmed plots in both RI and HI treatments. The comparison of the rarefaction curve (Supplementary Figure 2) showed a similar result to the diversity index (**Figure 5A**) and species richness (Chao1, Supplementary Table S3). PCoA analysis (weighted UniFrac) of the bacterial community for the control and warmed plots showed that the difference between bacterial communities in the warmed and the control plots was less in HI than in RI treatment (**Figure 5B**). In RI, the bacterial community was clearly different between warmed and control plots at 5 cm soil depth; the difference was bigger in the 5 cm than at 10 and 20 cm soil depth. These results suggested that the dissimilarity in bacterial community caused by warming could be overridden by increased irrigation.

## Discussion

### Microbial Community Responding to Soil Warming

Temperature has long been known a determinant for the growth and physiology of microorganisms and may be a determining factor for niche space competition among physiologically similar organisms (Sheik et al., 2011). The microbial communities analyzed in this study showed that the relative abundance of Actinobacteria, Bacteroidetes, and TM7 bacteria was positively correlated with soil warming, whereas that of Proteobacteria, Acidobacteria, Chloroflexi, Firmicutes, Nitrospirae, and Verrucomicrobia was negatively correlated to soil warming under both irrigation treatments, except that Bacteroidetes, Chloroflexi, and Firmicutes showed opposite trend with warming under high irrigation (**Figure 3** and Supplementary Table S2). The increase of Actinobacteria and decrease of Acidobacteria in relative abundance with soil warming were in agreement with the previous observations (Kuffner et al., 2012; Rui et al., 2015). The response of Actinobacteria to warming might be related to spore-forming ability which could be an advantage over other phyla likely to persist in warmed drier soil (Hayden et al., 2012). Actinobacteria are among the most important litter decomposers (k-selected) in soil and might be favored for soil organic matter (SOM) decomposition over other microbes because of adaptation to warming (Kopecky et al., 2011). Warming significantly increased the relative abundance of *Actinomycetales* (dominant order) and *Arthrobacter* (dominant genus) (Supplementary Table S2) which were previously reported their involvement in SOM decomposition and recalcitrant carbon degradation, respectively (Ferreira et al., 2008; Bengtson et al., 2012). The Proteobacteria and Acidobacteria showed lower abundance in warmed plot compared to control plots. A decrease in Proteobacterial phyla in warmed plot might to due to drier soil environment caused by soil warming, as Proteobacterial phyla found more responsive to wet environment than dry environment (Castro et al., 2010). Among the Proteobacteria, the relative abundance of Deltaproteobacteria (order Syntrophobacterales) and order Rhodospirillales (Alphaproteobacteria) was significantly decreased in response to soil warming, which could be due to soil drier condition in warmed plot than in control plots. Previous studies also reported the significant decrease in the relative abundance of order Rhodospirillales in response to long-term soil warming (Deslippe et al., 2012; DeAngelis et al., 2015). Acidobacteria are generally considered as oligotrophic organisms which grow well in lower carbon availability (Fierer et al., 2007). Previous study reported higher relative abundance of Acidobacteria in dry environment than wet environment, as dry environment slowed turnover of carbon in the particulate organic matter pool, which can reduce substrate availability and lead to more oligotrophic conditions (Garten et al., 2009; Castro et al., 2010). This is in contrast with our result, as we have found lower Acidobacteria abundance in warmed plot than in control plot. The decrease in abundance of Acidobacteria in our study might be due to higher dominance of Actinobacteria as these two groups are likely to share similar niches (Sheik et al., 2011). The phyla Gemmatimonadetes and Verrucomicrobia were lower and TM7 was higher in relative abundance in the warmed plot than in the control (**Figure 3** and Supplementary Table S2). The scarcity of the cultured representative of Gemmatimonadetes and Verrucomicrobia and TM7 phyla makes it difficult to ascertain their anticipated role in the ecosystem. However, owing to their significantly prompt response to warming, further research on their ecology and role in the environment is necessary. Our result indicated that different species might respond to climate warming at different rates and in different directions, resulting in an increase or decrease in the relative abundance of certain taxa.

Warming showed stronger effects on bacterial abundance, bacterial diversity, and community structure at surface layer than subsurface soil layers. The bacterial abundance (16S rRNA gene copy numbers) from the most of the samples was decreased slightly in response to warming. Other studies have also reported the decrease in bacterial abundance in response to warming in soil (Allison and Treseder, 2008; Castro et al., 2010; Hayden et al., 2012). Warming decreased bacterial diversity compared to control plot. The decrease in bacterial diversity might be due to the warmer and drier soil environment; it has been reported that warming treatment and soil water content strongly influenced bacterial population size and diversity in grassland soil (Sheik et al., 2011). The greater effect of warming on structuring of bacterial communities at surface layer (5 cm) than subsurface layers (10 and 20 cm soil depth) (**Figure 5B**) suggests that the effect of warming declined with soil depth. A declining effect of soil microbial communities over depth has also been noted by others (Rinnan et al., 2007; Deslippe et al., 2012).

### Nitrifiers Responding to Soil Warming

For nitrifier abundance, AOB abundance showed a significant response to warming when compared with AOA. Although the warming caused a drier condition and that condition could be restrictive to AOB growth (Chen et al., 2013), the AOB abundance was higher in warmed plot than in control, which might be due to the adaptive tendency of AOB to drier conditions. This result supported by Xu et al. (2016), who showed that under the simulated warmer and drier condition, the AOB community displayed rapid and significantly higher growth rate than that of AOA, with the population abundance being one order of magnitude higher than the control. Moreover, the previous study found that soil warming for +0–5 °C significantly increased the AOB abundance under N fertilization in boreal forest soil (Long et al., 2012). As in our study, warming that increased the soil temperature by 1.6 °C would significantly affect AOB abundance. Previous studies reported the soil water content and temperature could influence microbial activity (nitrification rate) and nitrifying community structure in soil (Avrahami and Bohannan, 2007; Gleeson et al., 2008; Tourna et al., 2008; Szukics et al., 2010). In our study, though the gene abundance of ammonia oxidizing bacteria was higher in the warming treatment, the relative abundance of *Nitrospira* (nitrite oxidizing bacteria) was significantly lower in the warmed plots compared to the control, especially at the surface layer (Supplementary Table S2). In the previous study, Rui et al. (2015) also found a low abundance of *Nitrospira* at a high temperature. The lower abundance of *Nitrospira* could be due to its sensitivity to drier conditions and, also outcompetition with other AOB species (i.e., *Nitrosomonas*) under high oxygen and substrate (NO_3_^-^) concentrations (Xu et al., 2016). Compared with AOB, the relative abundance of AOA was slightly lower in the warmed than the control plots. However, the AOA abundance substantially increased under HI irrigation, which means that AOA community was more responsive to high water content. Szukics et al. (2010) also reported that the AOA community rapidly adapted to high water content and lower temperature, while AOB community increased with increasing temperature. The decreased ratio of AOA to AOB in the warmed plot was mainly due to the increase in AOB growth at elevated soil temperature. The previous study also indicated that the ratio of AOA to AOB significantly decreased under the warmer and drier condition (Xu et al., 2016), which corroborated that the AOB community more rapidly adapted to the warmer and drier condition than did AOA community.

### Denitrifiers Responding to Soil Warming

Our study observed a significantly higher nitrate concentration in the warming treatments. Despite the higher nitrate concentration, the denitrifier abundance was lower in warming than in control plot. The decrease in denitrifier abundance might be due to drier soil condition produced through soil warming (Keil et al., 2015). Higher temperature leads to a higher evapotranspiration demand and thus a higher vapor pressure deficit, which could produce drier soils (Liu et al., 2013). The previous study concluded the reduction in soil water content by soil warming produces an oxic condition, which may be unfavorable for denitrification activity (Liu et al., 2016). A previous study reported that the low moisture and a higher oxygen concentration inhibited activities of denitrification enzymes in the soil (van Spanning et al., 2007). Warming increased nitrate and ammonium concentration than in control, which is consistent with previous observations (Liu et al., 2016). Xu et al. (2016) reported lower denitrification activity from warmed soil even though there was higher nitrate and ammonium concentrations compared to the control treatment, and concluded that warming could accelerate N mineralization and nitrification processes, not denitrification. This was consistent with our results of higher nitrifier abundance and lower denitrifier abundance in response to soil warming. These results were also in agreement with previous study by Barnard et al. (2006), who found that soil drying due to warming lowered the denitrification potential in soil. The denitrification in soil is mainly performed by facultative aerobic heterotrophic bacteria from diverse phylogenetic branches; therefore, it is hard to draw general conclusions about how warming influence the denitrifier composition, in spite of that the typical denitrifier genus *Bacillus* (Verbaendert et al., 2011) was found decreased with compared to control in the relative abundance in HI. Therefore, a combined higher temperature and drier scenario may affect denitrifier activity and/or abundance in soil.

The microbiome is an integral part of the soil, which is important for maintaining ecosystem function. A sound understanding of how these microbial communities respond to disturbances such as climate warming is limited. The study on how projected climate warming affects soil nitrifiers and denitrifiers community from wheat field soil is critically important for managing and minimizing the impact of climate change. The results from this study clearly demonstrated that the simulated temperature rise and drier soil condition could affect both the population abundance of nitrifiers and denitrifiers and the total bacterial community structure in soil. In summary, our observation revealed that experimental warming increased the soil temperature and decreased the soil moisture. Increased temperature significantly increased the PNR activity, NO_3_^-^ concentration, and AOB abundance in the soil but decreased (not significantly) the AOA abundance. Warmer and a drier soil condition tended to reduce the denitrifier abundance. Warming decreased the bacterial diversity and species richness and enhanced the relative abundance of species that have key roles in the decomposition of SOM. In RI, warming clearly yielded a significant shift in the microbial community structure, compared with HI, whereas the application of higher irrigation overrode the warming effect on the microbial community structure. Furthermore, warming had a pronounced effect on the microbial community structure at the surface layer (5 cm) compared with the deep soil layers (10 and 20 cm soil depths). Taken together, these results suggest that a projected warmer and drier climate change scenario would alter the population abundance of nitrifiers–denitrifiers and the microbial community structure (especially at surface layer), which, in turn, could affect the nitrogen turnover in the agricultural ecosystems.

## Author Contributions

TW, CH, and BL: conceived and designed the experiments. TW: performed the experiments. TW, SC, RS, and JL: analyzed the data. TW: wrote the paper. RS, CH, and BL: provided comments and improvements to the paper.

## Conflict of Interest Statement

The authors declare that the research was conducted in the absence of any commercial or financial relationships that could be construed as a potential conflict of interest.

## Footnotes

1 http://www.bioinformatics.babraham.ac.uk/projects/fastqc/

2 http://hannonlab.cshl.edu/fastx_toolkit/
