# Supplementary material for: Response of Nitrifier and Denitrifier Abundance and Microbial Community Structure to Experimental Warming in an Agricultural Ecosystem
Source: Front Microbiol. 2018 Mar 14;9:474. doi: 10.3389/fmicb.2018.00474 (PMC5861319; doi:10.3389/fmicb.2018.00474)
Supplement: Supplementary file 1 [file Table_1.docx]

**Table 1** Output of two-way analysis of variance (ANOVA) for nitrifier (AOB /AOA), denitrifier (*nirS*, *nirK*, *nosZ*) and bacterial 16S gene copy number. The table shows level of significance for abundance of archaeal/bacterial *amoA* genes, *nirS* genes, *nirK* genes, *nosZ* genes and total bacterial genes at different soil depth as affected by irrigation (I), warming (W), and their interactions. ‘*’, ‘**’, ‘***’ indicates significance level at *p* ≤ 0.05, *p* ≤ 0.01, and *p* ≤ 0.001 respectively, ns is non-significant.

|  | AOA gene copy number  (g^-1^ dry soil) | | | AOB gene copy number  (g^-1^ dry soil) | | | *nirS* gene copy number  (g^-1^ dry soil) | | | *nirK* gene copy number  (g^-1^ dry soil) | | | *nosZ* gene copy number  (g^-1^ dry soil) | | | Bacterial 16S gene copy number  (g^-1^ dry soil) | | |
| --- | --- | --- | --- | --- | --- | --- | --- | --- | --- | --- | --- | --- | --- | --- | --- | --- | --- | --- |
| Source of variation/  Soil depth (cm) | 0-5 | 5-10 | 10-20 | 0-5 | 5-10 | 10-20 | 0-5 | 5-10 | 10-20 | 0-5 | 5-10 | 10-20 | 0-5 | 5-10 | 10-20 | 0-5 | 5-10 | 10-20 |
| I | *** | *** | *** | ns | ns | ns | *** | * | *** | *** | ** | * | ** | ** | ns | *** | *** | *** |
| W | ns | ns | ns | *** | ns | ns | ns | ** | *** | * | ** | * | * | ** | * | ns | ns | ns |
| I x W | ns | ns | ns | * | ns | ns | ns | ns | * | ns | ns | ns | ns | ** | ns | ns | ns | ns |
