# Supplementary material for: Response of Nitrifier and Denitrifier Abundance and Microbial Community Structure to Experimental Warming in an Agricultural Ecosystem
Source: Front Microbiol. 2018 Mar 14;9:474. doi: 10.3389/fmicb.2018.00474 (PMC5861319; doi:10.3389/fmicb.2018.00474)
Supplement: Supplementary file 2 [file Table_2.docx]

**Table 2** Relative abundance (% of total reads) of abundant bacterial groups at the phylum (a), class (b), order (c) and at genus (d) levels. Different letters in same row are significantly different at *P*<0.05 (Tukey’s HSD post hoc test). Values with red color were significantly different than not labeled in each soil depth between warmed and control plots. Statistical comparison was made between warmed and control treatment for each soil depth under RI and HI irrigation treatment. W, warmed; C, control. Yellow shade indicates a warmed plot having significantly higher relative abundance than in control plot, gray shade indicates a warmed plot having a significantly lower relative abundance than in control plot.

|  | Regular Irrigation | | | | | | High Irrigation | | | | | |
| --- | --- | --- | --- | --- | --- | --- | --- | --- | --- | --- | --- | --- |
| **Soil depth** | 0-5 cm | | 5-10 cm | | 10-20 cm | | 0-5 cm | | 5-10 cm | | 10-20 cm | |
| **Treatment** | W | C | W | C | W | C | W | C | W | C | W | C |
| **a) Phylum** |  |  |  |  |  |  |  |  |  |  |  |  |
| Acidobacteria | 7.03b | **14.2a** | 10.5a | 13.4a | 12.4a | 14.7a | 8.63B | **11.5A** | 9.87A | 12.3A | 11.0A | 11.5A |
| Actinobacteria | **22.1a** | 18.6b | **22.4a** | 18.1b | **19.5a** | 15.1b | **27.9A** | 22.03B | **26.5A** | 20.9B | 21.4A | 20.0A |
| Bacteroidetes | **6.90a** | 5.30b | **5.45a** | 4.59b | 4.12a | 3.42a | 3.54A | 3.87A | 3.43A | 4.08A | 3.18A | 4.01A |
| Chloroflexi | 3.89b | **6.49a** | 5.74a | 6.80a | 7.64b | **8.49a** | 7.79A | 7.86A | 8.61A | 8.32A | 10.2A | 9.28A |
| Cyanobacteria | **5.06a** | 1.03b | 1.06a | 1.26a | 0.29a | 0.21a | 3.73A | 3.26A | 0.66A | 0.82A | 0.29A | 0.38A |
| Firmicutes | 2.02a | 1.98a | 2.15a | 1.99a | 1.94a | 1.99a | 1.61B | **2.35A** | 2.02B | **2.41A** | 1.70B | **2.19A** |
| Gemmatimonadetes | 3.39b | **5.39a** | 5.91a | 5.53a | 6.74a | 6.63a | 2.73B | **3.66A** | 3.31B | **3.87A** | 4.99A | 4.43A |
| Nitrospirae | 0.60b | **1.52a** | 1.33a | 1.50a | 1.96a | 2.32a | 1.17A | 1.51A | 1.42A | 1.54A | 2.13A | 1.95A |
| Planctomycetes | 5.10b | **8.58a** | 7.87a | 8.94a | 8.35b | **10.1a** | 7.12A | 8.50A | 8.34A | 9.24A | 9.68A | 9.04A |
| Proteobacteria | 24.6a | 25.6a | 24.1a | 25.8a | 24.3a | 25.5a | 25.1A | 24.8A | 25.1A | 25.2A | 23.8A | 25.9A |
| TM7 | **11.3a** | 2.1b | **4.3a** | 1.1b | **3.0a** | 0.5b | 1.8A | 1.9A | 1.2A | 1.2A | 1.0A | 0.8A |
| Verrucomicrobia | 1.30b | **2.14a** | 1.94b | **2.51a** | 1.55b | **2.12a** | 1.84A | 1.92A | 1.97A | 2.11A | 1.97A | 1.90A |
|  |  |  |  |  |  |  |  |  |  |  |  |  |
| **b) Class** |  |  |  |  |  |  |  |  |  |  |  |  |
| Acidobacteria-6 | 3.14b | **8.07a** | 5.72b | **7.39a** | 6.81a | 7.93a | 5.28A | 6.15A | 4.70A | 6.71A | 5.87A | 6.01A |
| Acidobacteria-5 | 0.047b | **0.18a** | 0.14a | 0.15a | 0.19b | **0.28a** | 0.10B | **0.16A** | 0.12B | **0.20A** | 0.16A | 0.18A |
| Chloracidobacteria | 1.36b | **3.06a** | 2.61a | 3.16a | 2.28a | 2.77a | 1.57B | **2.54A** | 2.18A | 2.45A | 1.92A | 2.29A |
| Solibacteres | 0.29b | **0.73a** | 0.51b | **0.75a** | 0.74a | 0.87a | 0.47B | **0.72A** | 0.59B | **0.87A** | 0.71A | 0.91A |
| Actinobacteria | **14.4a** | 6.99b | **12.1a** | 7.93b | **8.90a** | 4.97b | **14.9A** | 10.3B | **12.6A** | 9.68B | 9.04A | 8.53A |
| Cytophagia | **4.64a** | 2.89b | **3.02a** | 2.31b | 1.77a | 1.66a | 1.60A | 1.80A | 1.67A | 2.13A | 1.73A | 1.93A |
| Sphingobacteriia | **1.88a** | 1.23b | **1.20a** | 0.88b | 0.77a | 0.47a | 0.84A | 0.65A | 0.69A | 0.65A | 0.46A | 0.47A |
| Bacilli | 26.2a | 23.5a | 23.7a | 18.7a | 18.4a | 19.9a | 17.5A | 21.2A | 17.9A | 21.9A | 16.3A | 19.0A |
| Gemmatimonadetes | 1.31b | **2.26a** | 2.64a | 2.51a | 2.43a | 2.45a | 1.06A | 1.23A | 1.41A | 1.33A | 1.48A | 1.42A |
| Nitrospira | 0.7b | **1.52a** | 1.33a | 1.50a | 1.96a | 2.32a | 1.17B | **1.59A** | 1.42A | 1.54A | 2.13A | 1.95A |
| Alphaproteobacteria | 11.8a | 12.1a | 10.8a | 11.0a | 8.7a | 8.7a | 12.8A | 13.2A | 12.2A | 11.9A | 10.3A | 10.9A |
| Betaproteobacteria | 7.0a | 6.0a | 6.0a | 7.1a | 6.9a | 7.4a | 5.5A | 4.4A | 6.0A | 5.4A | 5.0A | 6.0A |
| Gammaproteobacteria | 3.6a | 3.8a | 4.1a | 3.7a | 4.8a | 4.8a | 3.4A | 3.8A | 3.4A | 4.0A | 4.0A | 4.5A |
| Deltaproteobacteria | 2.1b | **3.6a** | 3.2b | **4 .0a** | 3.8a | 4.6a | 3.4A | 3.4A | 3.6A | 3.9A | 4.3A | 4.4A |
| Phycisphaerae | 2.09b | **3.50a** | 3.16b | **3.70a** | 2.83b | **3.23a** | 2.81A | 3.37A | 2.97B | **4.75A** | 2.72A | 2.85A |
| Planctomycetia | 2.32b | **4.73a** | 4.48a | 4.69a | 4.17b | **5.23a** | 4.39A | 4.75A | 4.84A | 5.38A | 4.97A | 5.09A |
| Verrucomicrobiae | 0.11a | 0.14a | 0.12a | 0.24a | 0.08b | **0.17a** | 0.17A | 0.18A | 0.20A | 0.18A | 0.20A | 0.19A |
|  |  |  |  |  |  |  |  |  |  |  |  |  |
| **c) Order** |  |  |  |  |  |  |  |  |  |  |  |  |
| Solibacterales | 0.29b | **0.72a** | 0.51b | **0.75a** | 0.73a | 0.86a | 0.47B | **0.72A** | 0.58B | **0.87A** | 0.70A | 0.91A |
| Actinomycetales | **14.3a** | 6.8b | **11.9a** | 7.7b | **8.7a** | 4.8b | **14.5A** | 10.0B | **12.3A** | 9.4B | 8.7A | 8.2A |
| Rubrobacterales | 0.32b | 0.33b | 0.41b | 0.44b | 0.34b | 0.30b | 0.65A | 0.63A | 0.72A | 0.55A | 0.53A | 0.62A |
| Cytophagales | **4.05a** | 2.48b | **3.02a** | 2.31b | 2.27a | 1.66a | 1.60A | 1.80A | 1.67A | 2.13A | 1.59A | 1.93A |
| Sphingobacteriales | **1.57a** | 1.06b | **1.20a** | 0.88b | 0.77a | 0.47a | 0.74A | 0.65A | 0.69A | 0.65A | 0.58A | 0.56A |
| Nitrospirales | 0.60b | **1.52a** | 1.33a | 1.50a | 1.96a | 2.32a | 1.17B | **1.51A** | 1.42A | 1.54A | 2.13A | 1.95A |
| Rhizobiales | 2.95a | 2.96a | 2.97a | 2.92a | 3.22a | 3.06a | 4.25A | 4.22A | 4.02A | 4.01A | 4.17A | 4.30A |
| Rhodospirillales | 2.35b | **3.49a** | 3.28a | 3.29a | 2.85a | 3.07a | 4.78A | 4.11A | 4.69A | 4.25A | 3.37A | 4.03A |
| Burkholderiales | **5.23a** | 2.4b | 2.72a | 2.97a | 3.02a | 2.16a | 2.98A | 1.71A | 2.99A | 1.96A | 2.86A | 2.45A |
| Syntrophobacterales | 0.43b | **0.99a** | 0.80a | 1.0a | 1.13a | 1.39a | 0.70A | 0.89A | 0.84A | 1.08A | 1.01A | 1.20A |
| Xanthomonadales | 3.7a | 3.0a | 3.41a | 2.91a | 3.62a | 3.55a | 2.90A | 2.98A | 2.76A | 3.28A | 3.06A | 3.31A |
| Pirellulales | 1.38b | **2.36a** | 2.17a | 2.37a | 2.10a | 2.42a | 2.11A | 2.41A | 2.44B | **2.69A** | 2.61A | 2.44A |
| Gemmatales | 0.75b | **1.47a** | 1.38a | 1.47a | 1.48a | 1.77a | 1.29A | 1.54A | 1.59A | 1.79A | 1.73A | 1.65A |
| Planctomycetales | 0.34a | **0.58a** | 0.56a | 0.63a | 0.56b | **0.72a** | 0.48B | **0.63A** | 0.52A | 0.64A | 0.58B | **0.69A** |
| Verrucomicrobiales | 0.05b | **0.14a** | 0.09b | **0.27a** | 0.085b | **0.17a** | 0.22A | 0.18A | 0.20A | 0.18A | 0.20A | 0.19A |
|  |  |  |  |  |  |  |  |  |  |  |  |  |
| **d) Genus** |  |  |  |  |  |  |  |  |  |  |  |  |
| Arthrobacter | **1.22a** | 0.14b | **0.60a** | 0.14b | **0.44a** | 0.077b | **0.56A** | 0.29B | **0.40A** | 0.20B | 0.15A | 0.13A |
| Rubrobacter | **0.19a** | 0.15b | 0.22a | 0.17a | 0.15a | 0.10a | **0.31A** | 0.25B | **0.36A** | 0.17B | 0.22A | 0.27A |
| Agromyces | **0.18a** | 0.11b | 0.10a | 0.10a | 0.10a | 0.069a | 0.21A | 0.19A | 0.20A | 0.15A | 0.19A | 0.20A |
| Cellulomonas | **0.10a** | 0.034b | **0.044a** | 0.028b | 0.024a | 0.009a | 0.048A | 0.040A | **0.059A** | 0.019B | 0.037A | 0.015A |
| Actinoplanes | 0.21a | 0.054a | 0.061a | 0.025a | 0.035a | 0.025a | 0.31A | 0.066A | 0.074A | 0.15A | 0.025A | 0.049A |
| Saccharothrix | **0.04a** | 0.027b | 0.083a | 0.10a | 0.058a | 0.036a | 0.085A | 0.14A | 0.12A | 0.16A | 0.052A | 0.21A |
| Rhodocytophaga | **0.14a** | 0.02b | 0.043a | 0.026a | 0.03a | 0.02a | 0.048A | 0.057A | 0.012A | 0.018A | 0.002A | 0.009A |
| Bacillus | 0.88a | 1.19a | 1.34a | 1.16a | 1.18a | 1.17a | 0.95B | **1.52A** | 1.11B | **1.30A** | 1.02B | **1.35A** |
| Cohnella | **0.37a** | 0.081b | **0.22a** | 0.022b | **0.048a** | 0.027b | **0.078A** | 0.009B | 0.025A | 0.022A | 0.019A | 0.012A |
| Nitrospira | 0.28b | **0.64a** | 0.71a | 0.76a | 0.80a | 1.07a | 0.37B | **0.81A** | 0.74A | 0.70A | 0.96A | 0.83A |
| Mycoplana | **0.36a** | 0.08b | 0.085a | 0.039a | **0.058a** | 0.014b | 0.07A | 0.11A | 0.028A | 0.043A | 0.016A | 0.03A |
| Balneimonas | **0.23a** | 0.10b | 0.16a | 0.14a | 0.11a | 0.05a | **0.35A** | 0.18B | 0.19A | 0.20A | 0.10A | 0.14A |
| Rhodoplanes | 0.39b | **0.77a** | 0.69b | **0.77a** | 0.84a | 0.95a | 0.91A | 0.89A | 0.86A | 0.89A | 0.98A | 0.99A |
| Janthinobacterium | **1.57a** | 0.34b | 0.89a | 0.84a | **1.10a** | 0.59b | **0.86A** | 0.30B | **0.98A** | 0.55B | **1.18A** | 0.75B |
| Planctomyces | 0.34b | **0.58a** | 0.56a | 0.63a | 0.56b | **0.72a** | 0.48A | 0.63A | 0.52A | 0.64A | 0.58A | 0.69A |
| Pirellula | 0.24b | **0.42a** | 0.45a | 0.44a | 0.44a | 0.52a | 0.30A | 0.43A | 0.37A | 0.45A | 0.47A | 0.44A |
| Gemmata | 0.20b | **0.37a** | 0.41a | 0.42a | 0.35a | 0.48a | 0.27B | **0.40A** | 0.34A | 0.45A | 0.34A | 0.36A |
| Pseudomonas | 0.12a | 0.10a | 0.11a | 0.089a | 0.53a | 0.22a | 0.04A | 0.10A | 0.014A | 0.064A | 0.18A | 0.14A |
| Lysobacter | **0.36a** | 0.12b | 0.13a | 0.094a | 0.17a | 0.10a | 0.09A | 0.12A | 0.095A | 0.097A | 0.10A | 0.094A |
| Rubrobacter | 0.19a | 0.15a | **0.22a** | 0.17b | **0.15a** | 0.10b | 0.31A | 0.25A | **0.36A** | 0.17B | 0.22A | 0.27A |
| Adhaeribacter | 1.16a | 0.25a | 0.54a | 0.12a | 0.29a | 0.032a | 0.26A | 0.13A | 0.17A | 0.07A | 0.078A | 0.028A |
| Pontibacter | 0.45a | 0.60a | 0.29a | 0.29a | 0.23a | 0.050a | 0.21A | 0.43A | 0.14A | 0.23A | 0.053A | 0.074A |
| Devosia | **0.48a** | 0.20b | 0.29a | 0.24a | **0.29a** | 0.17b | 0.33A | 0.34A | 0.35A | 0.32A | 0.35A | 0.46A |
| Agrobacterium | **0.24a** | 0.044b | 0.093a | 0.046a | **0.085a** | 0.018b | 0.064A | 0.16A | 0.07A | 0.04A | 0.054A | 0.054A |
| Sphingomonas | **0.26a** | 0.15b | 0.18a | 0.19a | 0.086a | 0.047a | 0.19A | 0.20A | 0.13A | 0.11A | 0.063A | 0.073A |
| Phormidium | 0.48a | 0.055a | 0.47a | 0.39a | 0.03a | 0.04a | 1.88A | 1.00A | 0.14A | 0.24A | 0.03A | 0.11A |
| Pedobacter | 0.23a | 0.11a | 0.18a | 0.11a | 0.11a | 0.035a | 0.13A | 0.17A | 0.14A | 0.074A | 0.088A | 0.10A |
| Paenibacillus | 0.40a | 0.12a | 0.17a | 0.11a | 0.14a | 0.16a | 0.29A | 0.12A | 0.18A | 0.13A | 0.12A | 0.16A |
| Steroidobacter | 0.24a | 0.48a | 0.48a | 0.42a | 0.34a | 0.37a | 0.45A | 0.51A | 0.52A | 0.46A | 0.32A | 0.42A |
