# Supplementary material for: Response of Nitrifier and Denitrifier Abundance and Microbial Community Structure to Experimental Warming in an Agricultural Ecosystem
Source: Front Microbiol. 2018 Mar 14;9:474. doi: 10.3389/fmicb.2018.00474 (PMC5861319; doi:10.3389/fmicb.2018.00474)
Supplement: Supplementary file 3 [file Table_3.docx]

**Table 3** Estimated number of observed OTUs (at 97% similarity), richness, and diversity (n=3, mean SE) for warming and control at different soil depth in regular (RI) and high (HI) irrigation treatment. Different letters in same column indicate statistically significant differences (*P*<0.05) between warming and control treatment according to Tukey’s HSD post hoc test at *P*<0.05. n, number of replicates for each treatment; SE, standard error of means; W, warmed; C, control treatment; OTUs, Operational taxonomic units; Shannon, Shannon diversity index.

| Irrigation | Soil depth (cm) | Treatment | OTU_97%_ | Richness and diversity index | |
| --- | --- | --- | --- | --- | --- |
|  |  |  |  | Chao1 | Shannon |
| RI | 0-5 cm | W | 5427 ±185a | 16794 ± 780a | 10.91 ± 0.23b |
|  |  | C | 6100 ± 263a | 19929 ± 1936a | 11.74 ± 0.08a |
|  | 5-10 cm | W | 6140 ± 96b | 20266 ± 373a | 11.57 ± 0.15a |
|  |  | C | 6569 ± 3.84a | 21940 ± 322a | 11.91 ± 0.00a |
|  | 10-20 cm | W | 6327 ± 29a | 21268 ± 526a | 11.78 ± 0.01a |
|  |  | C | 6253 ± 16a | 21146 ± 253a | 11.82 ± 0.01a |
| HI | 0-5 cm | W | 6247 ± 111A | 20899 ± 885A | 11.68 ± 0.10A |
|  |  | C | 6446 ± 9.86A | 21693 ± 139A | 11.86 ± 0.02A |
|  | 5-10 cm | W | 6445 ± 21B | 21830 ± 229A | 11.82 ± 0.03A |
|  |  | C | 6578 ± 34A | 22764 ± 257A | 11.95 ± 0.02A |
|  | 10-20 cm | W | 6362 ± 107A | 21030 ± 243A | 11.86 ± 0.06A |
|  |  | C | 6484 ± 73A | 22717 ± 1045A | 11.90 ± 0.02A |
