# Supplementary material for: Response of Nitrifier and Denitrifier Abundance and Microbial Community Structure to Experimental Warming in an Agricultural Ecosystem
Source: Front Microbiol. 2018 Mar 14;9:474. doi: 10.3389/fmicb.2018.00474 (PMC5861319; doi:10.3389/fmicb.2018.00474)
Supplement: Supplementary file 4 [file Data_Sheet_1.docx]

**
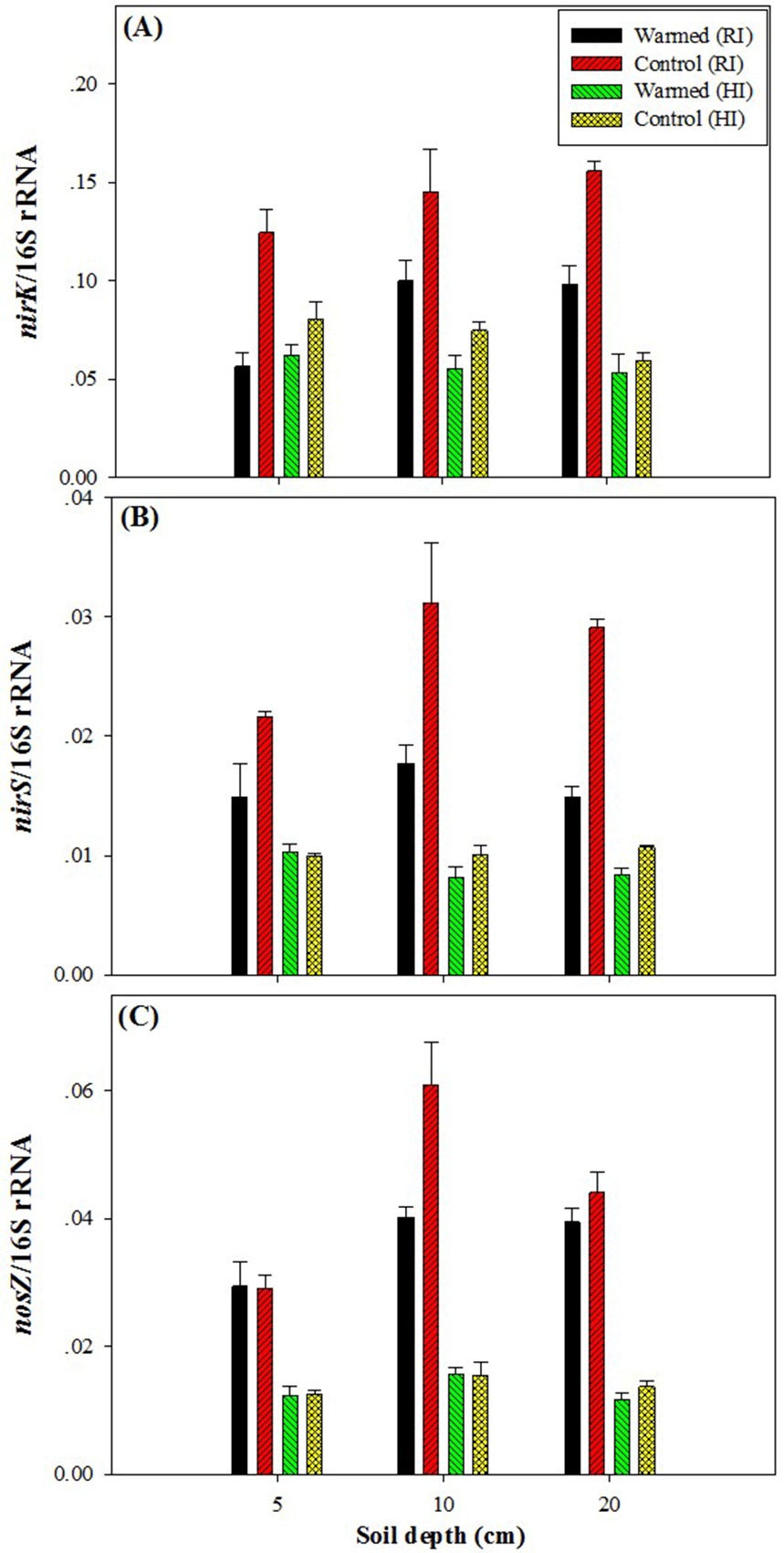
**

**Figure 1** The relative abundance of denitrification genes (A-C) in the warmed and control plots in regular (RI) and high (HI) irrigation treatment at different soil depth. Error bars indicate the standard error of the three replicates.

**Figure 2** Rarefaction curves for all treatments (A); warmed and control (B); and different soil depth (C).
